# Learning from disability studies to introduce the role of the individual to naturalistic accounts of disease

**DOI:** 10.1007/s11019-024-10216-9

**Published:** 2024-07-03

**Authors:** Ozan Altan Altinok

**Affiliations:** Center for Ethics and Law in the Life Sciences, Leibniz University of Hannover, Otto - Brener Straße 1, Hannover, Germany

**Keywords:** Disability studies, Evolutionary medicine, Niche construction, Patient autonomy, Extended evolutionary synthesis

## Abstract

Disability studies have been successfully focusing on individuals' lived experiences, the personalization of goals, and the constitution of the individual in defining disease and restructuring public understandings of disability. Although they had a strong influence in the policy making and medical modeling of disease, their framework has not been translated to traditional naturalistic accounts of disease. I will argue that, using new developments in evolutionary biology (Extended Evolutionary Synthesis [EES] about questions of proper function) and behavioral ecology (Niche conformance and construction about the questions of reference classes in biostatistics accounts), the main elements of the framework of disability studies can be used to represent life histories at the conceptual level of the two main “non-normative” accounts of disease. I chose these accounts since they are related to medicine in a more descriptive way. The success of the practical aspects of disability studies this way will be communicated without causing injustice to the individual since they will represent the individuality of the patient in two main naturalistic accounts of disease: the biostatistical account and the evolutionary functional account. Although most accounts criticizing the concept of disease as value-laden do not supply a positive element, disability studies can supply a good point for descriptive extension of the concept through inclusion of epistemic agency.

## Introduction: Concept of disease, value laden character and change; beyond value laden

From *drapetomania’s* horrible history (White 2008) to removal of homosexuality from Diagnostic and Statistical Manual of Mental Disorders [DSM] (Mendelson 2003) to varying degrees of success of medical interventions for health, medicine has a mixed record. While medical practice has increased the standard of living for many people, medical practices include paternalistic, sexist, racist, colonialist, and other kinds of biases as well as practices (Anderson 2006; Foucault 1975). To free itself from its troublesome past, one approach has been to exclude the negative aspects of the history of medical practice by defining disease as a value-natural way. Frameworks of normal or abnormal, or function and dysfunction require legitimization, and this is usually done either “positively” by naturalistic accounts such as (Boorse 1977; Millikan 1989; Nesse 2001a, b, 2007a) or “negatively” by “normative” accounts (Kingma 2014, Cooper 2020). Many scholars working within philosophy of medicine and philosophy of biology, most notably by after the works of Boorse (1975, 1977) Wakefield (1992), Millikan (1989), and Neander (1991) tried to ground a value free perspective. While the structural role of medicine was subjected to critique as creating a necessarily value laden concept of disease. Margolis (1976) sees the concept of disease similar to concepts of right or wrong in legal positivism, in the sense that these concepts are utilized in ways to intervene at a certain state of affairs by starting from choosing what is a desirable state and what is not, whereas Feinstein (1976) focuses on the “medical judgement” rather than concepts within medicine, or discipline of medicine in general to deal with different accounts of rule giving, whereas Canguilhem (1991 p. 196), argued that disease is only abnormal relative to a clearly defined context.

Be it value-free or not, the first aim of scholars working on the concept of disease as the initial reaction has been trying to define the concept. This has been tried by many, notably by the works of Nordenfelt (1986), Wakefield (1992), and more recently by Lemoine (2013). Based on the indicator they had at hand, different scholars did try to give a definition of disease or explicate it through conceptual analysis. One notable example of evolutionary definition of disease by leading philosophers of evolution as Griffiths & Matthewson (2018), where they try to subsume the concept of disease within an evolutionary definition of disorder. Among the evolutionary approaches, Nesse (2001a) discusses the difficulty of defining disease in general, and finding an evolutionary solution to give a collection of approaches that can provide a general perspective to define disease. Although these are helpful frameworks, they by themselves are also attempts to subsume the different concepts of disease, and various disease phenomena under a single concept of disease. Although the attempts of subsumption are not helpful, aspects of medical practice being informed by evolutionary views and perspectives can be tremendously helpful, as in diagnosis (Nesse 2007a).

There has been more nuanced argumentation, which can be exemplified by Kingma’s (2014) four-dimensional account in which naturalists and non—naturalists disagree on four different levels instead of the assumed “one”, while Broadbent (2019) uses his own two-layer distinction of value freeness and objectivity in classifying positions with respect to debates around disease. In Broadbent’s (2019) account, positions such as “value-dependent realism” can be found in works of Stempsey (2000) and Kass (1975) where value dependance is taken to be objective, in the sense that although they accept the concepts of health and disease to be value laden, they limit that judgement to good and bad. I will follow Broadbend’s (2019) understanding that the disagreements about value ladenness can be manifold, as well as his consideration of seeing diseases as secondary properties. What I will do will be, “naturalizing” the choices of reference classes and evolutionary histories with respect to the individuals’ autonomous actions and seeing disorder as a secondary property itself. From a pragmatic point of view, both objectivity and value ladenness are matters of convention. However, this is not simple conventions of blending different point of views, but accepting certain plurality of methods, results, and the set of entities. I will take objectivity as transparency, and I will claim that the agent in the making of her environment actively takes part in how she acts within her autonomy as well as how she should be contextualized.

## The components of the concept of disease in biopsychosocial model

Although in the mainstream debates of conceptual analysis the discussions around concepts and concept use have been ongoing to include not only a concept in isolation including only the intension and extension of a concept as in the traditional sense of Putnam (1975), but the concepts with their epistemic goals (Brigandt 2010, 2014), the arguments around definition have been quite influential. Now rather than focusing on the definition of the concept of disease alone, I briefly mention the “models” of health and disease, in which health and disease as concepts are classified within an established framework. This aspect is an important gate towards disability since it also operates as a “model”.

Engel (1977), within the mainstream philosophy of medicine, provided one of the first influential works within the field to build a structured modern understanding around medical practice with a multitude of set concepts, rather than working on the concept of disease itself. The talk of general models within medicine in this respect took its final shape in the form of the “Biopsychosocial Model” as it was the first generally accepted “model” in modern medicine. The aim of this model was to counter the dominance of “biological model”, which was claimed to be the decisive model of the time. Within the Biopsychosocial model, the concepts of health and disease were connected to social and psychological phenomena to include individual’s social relationships such as to family and to friends, and psychological mechanisms such as coping methods. Although it was subjected to change at different levels of engagement within practice and theory, and different elements of the model has been changing for quite some time, it replaced the “biological model” as the mainstream model of health and disease in their relationship to medicine in general both in theoretical and clinical aspects (Hofmann 2002).

The discussion around models instead of only concepts also enabled multiple perspectives from social and psychological domains to be constructed within their relationship to the individuals. The practical application, in the structure of patient related aspect in the making of concepts of health and disease, as put by Hofmann (2002) was to give at least some space for the patients’ experiences in the structure of the concept of disease, was the sickness (social domain), disease (expert/medical domain) and illness (personal/patient domain). Following the discussion that is mentioned in introduction, the critique of a “purely biological” concept of disease has been criticized on the value-based account of its own inclusion of the related concepts. The critique also goes through the biopsychosocial model. This leaves the concept of disease to be an emergent property realized within a certain interaction. Cooper (2020) argues, referring to the biopsychosocial model. “On the ‘medical model’, the disorder lies within the body of the wheelchair user. On the social model of disability, the problem is located within the environment” (p. 156).

To overcome this further difficulty of seeing health and disease distributed within the set of relationships, One Health Approach has been put forward. One Health Approach, since its development has been quite popular amongst recent philosophers of disease, particularly within disability studies, is also embedded in the policy making and the UN advisory framework (Zinsstag et al. 2020). In a form of introduction to One Health, Morar and Skorburg (2018) focus on distribution of the concept of health out of the organism, as they put it; “… develops and defends the Hypothesis of Extended Health (HEH), which denies the claim that health and disease states are predicated solely on the internal functioning of an organism. If this is correct, then the targets of medical invention and ethical concern are wider and more diverse than is usually assumed” (p. 341). They base their study on the various accounts of distributed understandings of cognitive science, social and personal psychology, neuroscience and microbiology. This distribution, while creating a good framework, can also be supported by evolutionary biology due to the lack of translation from “the models” to the entire framework. Although there has been an increase in the number and the methods of models, there has hardly been any translation back towards the concepts of disease and health themselves.

First, I will focus on a little summary of disability studies to point out the role of the individual, also claiming that there are attempts within disability studies itself to naturalize the social model. Second, I will point out EM and the discussion around proper functions to be enriched by the individual history, and thirdly I will investigate the biostatistical model to integrate niche construction and conformance.

## Disability studies’ contribution; relationship between the social and the biological

Disability studies, now a large field of its own, started off with a critique of the established views of health, such as the biostatistical model. Historically, disability had been perceived through a medical model that primarily focused on diagnosis, cure, and rehabilitation, often neglecting the social and cultural aspects of disability experiences (Shakespeare 2013). However, with the rise of the civil rights movement in the 1960s and 1970s, disability activists and scholars challenged the prevailing medical model and advocated for a paradigm shift in understanding disability as a social construct (Barton 1996). This marked the beginning of the social model of disability, which emphasized that disability is not solely a consequence of impairments but also a result of societal barriers and discrimination that hinder full participation and inclusion (Oliver 1990). The emergence of Disability Studies as a distinct academic discipline gained momentum in the late twentieth century. Pioneering scholars like Erving Goffman and Irving Zola critically examined the social construction of disability, influencing a generation of researchers (Barnes et al. 1999). In 1982, the Society for Disability Studies (SDS) was founded in the United States, and in 1992, the first international journal, “Disability & Society,” was launched, further institutionalizing the field (Barnes 2003).

Despite the successful history, there has been calls for “empirical sensibilization” of the models of disability. Although there are some different classifications like Gustavasson’s (2004) where he distinguishes models further as he puts the relationship between a person’s abilities and the functional demands of the environment coalesced in Scandinavia and Europe to the ‘Nordic Relational Model of Disability’. He characterizes this model as an empirically sensitive, multi-level approach that is both relative and interactionist. Many frameworks within disability studies rely heavily on the social model due to its multi-layer success, however the social model of disability has been criticized as well, for overlooking the medical and social dichotomy (Shakespeare 2006). Shakespeare further problematizes how social model has been insufficient to explain the lived experiences of the individuals with disability, and the expansion of the biological understandings are required on an individual basis to account for different instances of disability (2006). In a similar strand, the mainstream medical model of disease sees illness, sickness and disease related concepts through practical means (Hofman 2001, 2002) whereas disability studies sees the very concepts of disease, illness and sickness relational.

Amundson (1992), one of the trail blazers of disability studies within the “pre social model” framework makes the problem agenda of the “social model in the making”, visible.

“Disability restricts opportunity. How? The restriction seems to follow simply from the fact that disabilities involve *species-atypical function* together with the fact that species typical function is an especially efficient means of *procuring the goods available in the environment in which an individual of a particular species finds itself*. This answer seems obvious enough in view of the biological (or evolutionary) principle that members of species fit well into their environments. Functionally atypical species members simply won’t fit as well as typical members into the species’ typical environment. But, as always in biology, things are not this simple. Complications arise from the fact that the environment in which members of the human species pursue their goals is largely constructed by human beings themselves” (p. 109) (My italics)

The parallel of fittedness to the environment in evolutionary and individually is striking. About the first one, both historically and ahistorically, there have been studies about the fittedness debate in which different organisms could be fitted to some environments (Krohs 2022) as well as the groups evolving to fit non species normal functions. The developed version of his perspective following a relational model like the distinction between handicap and disability (Amundson 1992; Firth 2023) which is sees disease as a secondary property following Broadbent (2019) is the current understanding within disability studies. Although within the social model of disability, nor within the framework of “naturalistic accounts” this connection is not made, I argue that the disease vulnerability framework is a good extension of the secondary properties account, and niche construction accounts with construction/conformance/choice also are working within the same framework. Within the more traditional models of disease which makes it to the mainstream understanding of disease; Nordenfelt (1986, 1993) also mentions that the goals of the agent should be also considered in the understanding of disability.

In general, *relationality* is considered to be a common way of thinking about environment—individual interactions within disability studies. The novel element I introduce here is that, amongst the possible *relationships* of the species characteristic and possible environments are allowed, the individual can choose the one in which the individual can be in relationship. That is to say, since the individual cannot exist in isolation from the environment, the question becomes about the patient herself rather than the environment. My critique is about the difficulty of distinguishing between the social and the natural in many frameworks of biology. This critique is supported by the ahistorical (or less historical) understandings of niche construction and historical ones of evolutionary medicine. Although the distinction between the social and the biological was meaningful in creating the framework for disability studies (Shakespeare 2006), the domains where the biological is socialized, and the social is seen as a part of biology, evolutionary biology, and behavioral ecology could help to solve this problem. I would like to concentrate the following points of disability studies for this paper, although the field can be translated in many other aspects as well;

A) The individual “sets” certain goals and environments and hence has an epistemic autonomy to determine if a situation is a handicap or a disorder, whereas one of the most important properties is also the disorders being relational. However, unlike the critiqued views on the limitation of the “social aspect” and the “social model” I think it is possible to integrate individual agency and relationality to the existing concepts of disease within the main naturalistic models, which can be used by not only people of disability, but by everyone. I will deal with that in the niche construction part, arguing that Environments are constructed and the species typicality is not that strong of an argument anymore. There is no easy species typical environment, because of—in a way—niche construction.
B) There is no species normal, but possibilities of actualization as a goal within niche construction (being in a certain niche). The goal of the individual can be seen as occupying a certain space from the epistemic perspective of behavioral biology. Species specificity and goals of the species-specific networks within environmental behavioralist understanding allow naturalistic individualization. We can use species specificity for general diagnosis, but the final reference class can be structured. If we accept this dynamism, the discussion can be centered around how many of the goals of the agent are held from being realized in the context.

## Proper evolved functions within evolutionary medicine

My main aim here is to take the evolution of disease vulnerability perspective more seriously and combine this perspective with the emergent disease model of Broadbent (2019). Within evolutionary thinking, disease vulnerability as an ontological claim has been pointed out by Nesse (2007b, 2011). Although I will mostly highlight the uses around proper function here, it is possible to enrich the conceptual framework further by anyone who would like to develop the perspective in that direction.

One very important framework in which the concept of disease is naturalized is evolutionary function, or the etiological account. Sarto–Jackson (2018), following Millikan (1984, 1989) and Neander (1991) argues that the etiological theories of function are gaining a stronger ground within “non normative” theories of disease, making to the mainstream. Amongst etiological accounts, Wakefield’s “harmful dysfunction analysis” of disorder (Wakefield 1992, 2011) is the most cited one. The “harmful dysfunction analysis” employs a concept of function that is assigned to traits which explains their coming into existence and their stability throughout many generations in that species (Wright 1973). This account embraces an etiological conception of function, according to which functions are selected effects, and the function of a trait is the effect caused by the trait that explains the continued reproduction of past tokens of this trait. The main emphasis is the life history of the trait itself.

The immediate challenge of mitigating proper function accounts and contextual understandings of disability studies is the generalist, population-based conception of evolution of traditional EM. To counter this, I will use the disciplinary understanding, rather than only a particular section of evolutionary frameworks to talk about disease within an evolutionary context to enable a space of extension of evolutionary function. The challenging question when different inheritance mechanisms are involved is, what to do when proper functions do not exist in a multiply realizable plane? In the sense that, *a* exists as far as *b* from the previous generation, but through *d* in the latest generation. The other problem is, proper functions if we assume a good epigenetic account of inheritance of them, can never be final from several generations to the lifetime actions of the individual. Here, agent-based accounts of an individual’s own positioning of the self can be of help.

### Evolutionary medicine and disease

Evolutionary Medicine (EM), the most contemporary established approach (Zamperi 2009) in the attempt to connect medical thinking with evolutionary perspectives, provides a framework of disease which can be used to reconstruct the evolutionary history of organs, traits or tissues which could be used to give an account of function. Although EM is not the first attempt to approach medical practice through Darwinian or evolutionary thinking, it has gained a strong influence within departments of biology, evolution-related fields, and, to a certain extent in medical facilities to the extent that it had made its place within textbooks of medical faculties (Grunspan et al. 2018). There are strong attempts for institutionalization of EM in the contexts of medical education, not only explanation directly coming from evolutionary structures (Trevathan et al. 2008, Trevethan 2018). Stearns (2012) is more ambitious about the scope of evolutionary approaches within medicine, giving it a leading role instead of a supplimentary role.

At the core of the research programme of EM is the match—mismatch hypothesis. This is a framework which is not limited to but useful at understanding the “diseases of civilization”, (Williams & Nesse 1991; Nesse 2019) which are based on the idea that most diseases such as autoimmune disorders, certain mental disorders, many behavioral disorders and many others are due to a mismatch between the way current humans live, and the conditions to which they were adapted for, it is based on the idea of stable environment of Environment of Evolutionary Adaptedness (EEA) of Bowlby (1969) where most human traits and structures are selected for, which faced a big shift due to agricultural revolution. This is the general approach to structure the origin of the proper function of a trait or an entity within the etiological accounts of disease. Drawing on the recent research, as well as the historical accounts of EM, it can be easily said that EM has a framework that is causing a shift of focus within medical epistemology through its emphasis on disease vulnerability rather than disease itself (Altinok 2022). And it enables us to restructure the disease as an actualization between a certain kind of mismatch between both entities of environment and organism. Disease vulnerability, being more central due to its relationship to more evolutionary entities rather than entities which do not have that strong “historical reification” as they do, has stronger explanatory relevance. This perspective of EM is rooted heavily in the imagined ideal relationship between the organism and the environment in which the evolution within the EEA selected for certain traits, structures and mechanisms which are inherited within generations, and are supposed to be, contrasted with the other traits, within the general understanding of standard perspectives within evolution, have more explanatory relevance within the dichotomy of evolutionary and non-evolutionary biology (Altinok 2023).

Following Uller et al. (2020) against the standard evolutionary set point EEA, it is possible to try to reconstruct evolutionary explanations within the framework of EES (Extended Evolutionary Synthesis) in which they do not only take the processes of evolution out of the generic structure of MS (Modern Synthesis) but they also through empirical research mostly in the direction of evolutionary and developmental biology to include certain entities and mechanisms within evolution to operate as causes of evolution. This means, it will be possible to imagine the organism differently with various kinds of set of behavior and relationships within EM. When the imagination of the past takes place, which includes the disciplines of behavioral biology, in specific behavioral ecology, the results of such an imagination, and the construction of the concept of disease will be different based on disciplines used in particular imagination of the past. In short, the starting point of the metaphysics of disease is different in EM compared to the standard models with its focus on disease vulnerability structures.

### The critique of evolutionary medicine, dreaming of the past, how to reconstruct an EEA with behavior in mind

Here I will argue for multiple points of EEAs to create different etiological models, however not necessarily beyond natural selection, but beyond a stable point of selection which could be decided only through the action of the individual. A similar point is made by Satro-Jackson (2018) as “etiological considerations that go beyond the principles of natural selection have not yet or only marginally been integrated in most models of disease” (p. 33). Here, I will try to address this gap.

Evolutionary medicine provides a good framework particularly with respect to individualization of emergent token diseases as conditions. However, Cournoyea & Kennedy (2014) and Cournoyea (2018) argue that the explanatory relevance that is required by medical explanations is lacking in the explanations of EM. The critique goes in the direction of clinical relevance. According to the claim, evolutionary explanations in medicine are clinically not relevant. The ultimate explanations that EM provides are not helping patients clinically, or moving medical research in a positive direction, they claim. To a certain extent, with respect to the standard canon of evolutionary medicine this claim will hold true if we limit ourselves to explanations alone. However, the standard canon of evolutionary medicine has more to offer than only providing explanations, including providing research programmes, conceptual directions and research agendas (Altinok 2023). Impact of EM in making research programmes put aside, the new developments within medicine, which go beyond the proximate—ultimate causation assumes the proximate—ultimate distinction to hold within disciplinary distinction of evolutionary theory. Baedke (2017) discusses the possibility to expand causality within evolutionary theory beyond the usually held evolutionary causation mechanisms, and with his observation from the explanations that the evolutionary biologists use, he concludes that the causal explanations that evolutionary biologists use are going beyond the usual structures of Mayrian evolutionary explanation within Mayr’s proximate—ultimate distinction. This observation is in line with the general statement of Laland et al. (2011, 2014, 2015) as well as the earlier analysis on the theoretical decision of Müller & Pigliucci (2010), with respect to the reduced usefulness and even obstacle of the dichotomy within biology. Seen certain shortcomings of the traditional perspectives of evolutionary medicine, which are usually not very up to date with the recent research happening in evolutionary biology, including the research done on niche construction and individualization at the practical epistemic levels (Altinok 2022).

Against the limitations of MS, research on epigenetics is based mostly on the research tradition of Jablonka & Lamb (2005), Jablonka & Raz (2009) which emphasizes the importance of stability of inheritance mechanisms within a couple of generations. This centrality and multiplicity of mechanisms of inheritance made it well established also within the research structures within epigenetics (Baedke 2018). I claim that it is possible to restructure our view of EEAs based on localized EEAs. One theoretical limitation of EM is within its imagination of the structure of EEA. EM tends to stabilize certain traits with respect to the relationship between the individual and the environment. In the traditional accounts, the modern human (also “imagined” as mostly western, white and male etc.) is individuated as a stable biological entity from the modern world and then put within the assumed EEA.

If we accept the claim that instead of a singular EEA, we can think of many which can be constructed based on the manifold of epigenetic and related shorter term inheritance dynamics, the second question to be asked is, where are the boundaries of the individual? Through asking this question, it would be possible to extend the explanatory potential and the narrative of EEA, therefore making certain behaviors a part of the organism’s own existence and see the boundaries in which we should be reconstructing the individual in “isolation” and abstraction. Although not mentioned within a framework I have put forward, I think Nesse’s *Smoke Detector Principle* (2001b) coming from evolutionary psychiatry, or similar frameworks of explanation where a behavior or a set of behaviors within organism’s wellbeing are taken to be “isolated” with respect to whichever environment the organism is, are good candidates of such explanations.

Following Kranke’s (2022) definition of “historical explanations of type phenomena” which she sees to have the possibility of explaining multiply realizable phenomena, an evolutionary account of dysfunction or disease, observing the acceptance of pluralism of field (Griffiths & Matthewson 2018), evolutionary functions also need to consider the individual autonomy. If we accept that inheritance mechanisms are various for the “same” trait and the inheritance mechanism that is dominant to determine the proper function is based on the individual agency we can argue that; Dysfunction judgements (evolutionary medical judgements as well) are type judgements which categorize a certain individual, organ or behavior as a member of a certain framework. Even though they can be various and convincing they can not be conclusive (type token and dispositional—relational aspect) within clinical intervention. Following the mismatch to modern environments accounts (Nesse 2001a, b) it makes sent to accept when people (patients) categorize themselves through the causality regimes that they are categorized through interactions of the medical structure which are parts of their environment. Medical judgements can only be testable (not even tested) when the entire “disease” process is “completed”, in cases of medical intervention. The patient either agrees to the initial judgement of diagnosis and acts based on the assumed mismatch, or decides against based on some other mismatch constructed around her individual EEA (in cases of non-interventive medicine—such as a terminal condition, from epistemic to none) and puts herself in a different regime since her activities, and hence what she will be judged with in the future will be different.

As I pointed out in the previous part, one general domain of extension of the concepts of health and disease was about the interaction between the environment and the individual, which is the underlying theme of disability studies, as well as the “state of the art” version of the biopsychosocial model, One Health. If we aim for imagining an environment within the history of the species, or sub populations and even generation, together with the inheritance of the behavioral characteristics that they have, their ability to choose an environment has more to do with their relationship to themselves, that is to say, can be seen as an element of autonomy not only epistemically, but at the more material making of integrity.

## Niche; realized versus potential, reference class of biostatistical model

The other prominent (more than the evolutionary functional account) “naturalistic” account of disease is the Biostatistical model by Boorse. This account takes function as a ‘species-typical’ contribution to survival and reproduction (1976). Disease is defined as the failure to function according to a species design. This can occur through functional efficiency being either degraded below the typical level or limited by environmental agents (Boorse 1977; Tresker 2020). Boorse understands this as functioning “more than a certain distance below the population mean” (1977, p. 559) for the relevant set of humans. Since the account is rather old, and it has gained more nuance through the critiques; I would like to represent the latest account that he restructured (2014, p. 684):

1. The reference class is a natural class of organisms of uniform functional design; specifically, an age group of a sex of a species.
2. A normal function of a part or process within members of the reference class is a statistically typical contribution by it to their individual survival [or] reproduction.
3. Health in a member of the reference class is normal functional ability: the readiness of each internal part to perform all its normal functions on typical occasions with at least typical efficiency.
4. A disease [later, pathological condition] is a type of internal state which impairs health, i.e., reduces one or more functional abilities below typical efficiency.

Kingma (2014) argues that the selection of key parameters to operationalize health/function and dysfunction/disease is the important and the lacking element in BST. In addition to that a crucial role in Boorse’s account is played by reference classes, that is, the groups in which we can define the statistically normal function as normal function/health (Kingma 2007). Through niche construction it would be possible to “naturalize” the individual’s autonomy within reference classes, like that of disability studies. Although Boorse claims that the reference classes that are picked are matters of convention while retaining to be objective (1977), he does not make a point on who should decide on the correct reference class for the given individual or the groups of individuals, instead opting for the possibility of agreement eventually. Although I do not in principle object to the possibility of convergence towards an agreement, the process itself can be lengthy in making of the reference classes. In practice, the common way of delegating the decision is assuming that the experts will have the ability to judge the individual and find the correct reference classes. An individual in this sense should also be able to express her interest at least in each group.

Since in Boorse’s account, not all members of a species have the same design in every respect, we need to specify reference classes according to biologically relevant subgroups. The crucial question here is who decides on the appropriate group in which the organism/patient or a set of traits of the organism is to be evaluated. Arguments for the possibility of the patients’ selection of their own environment to assess the health and diseased situations, making the environment part of the patient autonomy in the process of disease definition and respective healing have been made separately. From disability studies, Nordby (2019) asks the question of epistemic authority about the “ownership” of the concepts of disease and sickness. In his analysis, he does not ask only how the concept can be defined but who can define it. He argues that in most cases, both concepts of disease and sickness are driven by medical experts, although the concept of sickness was reserved to be defined based on the patient experience of the “disease”.

I will argue below that, employing niche construction theory, that there is not only not a singular species normal, but the individuals are effectively choosing the kinds of reference classes to be evaluated in. Moreover, the necessity to introduce the individual agency and the individuals’ own selection of the environment is already given in niche construction theory, would be necessary to involve in choosing reference classes, “naturalizing” agency.

### Niche conformance and biostatistical model

I called into question uniform functional design and typical efficiency within Boorse’s account. Following the relational understanding of disease, the relation towards what is the next question, or in other words, within which environment should the organism be evaluated about the health and disease situation? I will claim that this relationality is connected to organismic activity and is related to the judgement of the individual. In this part I will argue that niche construction and conformance *are* individuals’ goals being associated within a particular kind of being which resembles “uniform functional design” and typicality of Boorse which I mentioned earlier.

The founder of the modern debate of niche construction Odling-Smee (1988) started to bring the organismic activities’ roles within evolutionary biology. Odling-Smee et al. (2003) argue that organisms actively alter both their own and one another’s evolutionary niches through the process of niche construction. Examples include how animals construct mounds, burrows, and other artifacts; how plants modify the physical and chemical environment; how shadows are created, affecting wind direction; and how plants modify the cycle of nutrients. Evolution via niche creation is a potential result when such adjustments change the demands of natural selection. Matthews et al. (2014) propose a set of criteria to test for the presence of niche construction: (a) organism must significantly modify environmental conditions; (b) Organism-mediated environmental modifications must influence selection pressures on a recipient organism; (c) there must be an evolutionary response in at least one recipient population caused by the environmental modification. These criteria are further developed by Laland et al. (2016); “(1) organisms modify environmental states in nonrandom ways, thereby imposing a systematic bias on the selection they generate, and allowing organisms to exert some influence over their own evolution (Odling-Smee et al. 2003; Laland et al. 2014); (2) ecological inheritance strongly affects evolutionary dynamics (Odling-Smee et al. 2003), and contributes to parent–offspring similarity (Danchin et al. 2011; Bonduriansky 2012; Badyaev and Uller 2009); (3) acquired characters and byproducts become evolutionarily significant by affecting selective environments in systematic ways, and (4) the complementarity of organisms and their environments (traditionally described as ‘adaptation’) can be achieved through evolution by niche construction (Odling-Smee et al. 2003)” (p. 192).

As can be seen, how they are selected for, and how it is related to fitness and the health of the individual within this framework is dependent on the environment that the individual “moves into”. The activities of the individuals with respect to their environment which takes part in the making of the typicality of the individual can be found in the literature of niche construction. However, these criteria are not the only ones, Trappes et al. (2021a, 2021b) also discuss that there are different concepts for niche construction. They also claim the existence of further individual-level ecological mechanisms in niche constructions, some of which are more prevalent for some individuals, under certain circumstances rather than a one size fits all model. The processes where the focal individual responds to their environments by performing a focal activity are defined further by them as; selecting a part of the environment in niche choice, altering the phenotype in response to the environment in niche conformance, or making changes to the environment in niche construction (Kaiser and Trappes 2021; Trappes et al. 2022).

### Individual within niche construction

Within the framework of individualized niche construction to extend the perspectives of research on patient autonomy in the making of their own environments I claim the following are fundamental; (a) determination of the disease situation based on the kind of epistemology that the patient is interested in and found herself (b) provid an autonomous stage for the possibility of constructing the desired environment for the patient at the material setting since the behavioral aspects which are the result of environment—individual interaction is determined by the individual at least to a certain extent. I take the niche to be the “effective environment to the individual” in the sense that the relationships the organism has with the niche are the strongest effects. From here, I will also assume that it is quite normal that organisms are actively constructing their own environments, which can be exploitered to patients’ conception within health related models. And it is also up to the organism to “know” which strategy to be the better one, which environment to exist is “up to” the organism. This “knowing” can be grounded in epigenetics and niche construction, scaffolding, embedding and the type—token actualization within the organismic affordances or niche affordances. I would suggest using two different reference classes in this regard, following the experiences from niche construction. A complete judgement of disease is not possible because of the failure of achieving this distinction. Because of that, a combined judgement for now with current reference class and the future with reference classes makes sense. Here lies the individualization problem.

One pressing issue about the departure is about making evolution more about individuals. Further research can be of help in terms of inheritance and thus relatively stability (Trappes 2021a calls this *robustness*). The concept of individuality as she uses is pluralistic, however her analysis further shows that biological individuality means that the inherent quality of differences makes sense in the definition of the concept. Moreover, robustness is seen as an intermediate between the long-lasting evolutionary processes and the current state for the most helpful strategy for the particular individual within natural selection. “That is, each individual’s ranges for the niche dimension are similar or corelated over time and in different contexts relative to other individuals’ ranges for the niche dimension” (p. 61). As can be seen here, it starts from a metaphysics of earlier understanding of the niche, and then realizes it within the population. As the organism itself is shaped by its own disease vulnerabilities in its past, it can still use the “species actualization” as the potential space of action. With respect to the Biostatistical model, in the analysis of niche construction theory the distinction between the fundamental and realized niche is also argued for (Trappes 2021a); “The fundamental and realized niche both depend crucially on the species’ phenotypic properties, which determine its resource use, distribution, and requirements and tolerance … On this view, the niche would be a relational property, consisting of a species’ possible or actual relation to ecological conditions that permit its persistence” (p. 56).

Similar to the relational understanding of disability, niche construction theory also sees a gap between actualized and the potential properties. The fundamental and realized niche both depend crucially on the species’ phenotypic properties, which determine its resource use, distribution, and requirements and tolerances. Partly for this reason, some authors characterize the niche as a species’ property (Schoener 1989; Kearney 2006; Pocheville 2015). On this view, the niche would be a relational property, consisting of a species’ possible or actual relation to ecological conditions that permit its persistence” (Trappes 2021a, p. 56). From a niche construction perspective, this could be seen as failure to the realization of some of the special potentials by the niche. I take these goals to be not only within the consideration of the medical personnel, but to see these goals’ fulfillment as the desired reference class, similar to that within “disability restricts opportunity” (Amundson 1992).

One detail to note here is that this is the phase when the theoretical set up is made to be filled, however epistemically determining what to be found by the researcher is still—naturally—decided by the researcher. This is new in the patient perspective. Already the ecological definition distinguishes itself from the evolutionary definition if niche by focusing on the individual rather than population. Gain and loss of function can be traced to properness (not actual benefit) only through following the individual.

## Conclusion

The perspectives of disability studies have been providing critique which were the driving force of change in the making of the concept of disease. I added some “mechanisms” as translations of these perspectives to the naturalized accounts of disease. This way, hopefully the frameworks of naturalisms can close the gap from having the principles only at the abstract level for disability studies to be able to have working mechanisms which can be applied to provide legitimization through biological vocabulary to give them mechanisms to operate with. This could prepare a patient-based, individualized emergent disease framework to be used within the naturalistic accounts.

Nordenfelt (2017) famously asks the question; “Health, disease, and the other central medical concepts are not used only in the human context. We ordinarily ascribe health and disease also to animals and plants. Do we then apply the same concept of health? In this case the answers differ. The naturalists, who relate health solely to survival and reproduction, can easily transpose their concept to the world of animals and plants (For such a view see, Broom 1993). The same could hold for balance theorists. It is more problematic to use the idea of health as ability or, even more, the idea of health as well-being all over the world of animals and plants” (pp. 39–40). In this sense, having an empirical agenda that can be translated from behavioral ecology can be of help in understanding patients’ relationships to the environment, satisfying the expectations of non-human contexts as well as larger models such as One Health.

If we can conceptualize changing Environments—individualization different conceptual frameworks, (trying to understand the differences of use for evolutionary biologists of different kinds to reach different aims) we can use persistence of individuality (and making of the borders of the individuality in this respect by the organism) in the changing environments to give us grounds to (a) research different epigenetics inheritance of individualities that are connecting behavior to inheritance (b) understand (through the added effect of a) individuality as a type in which we can reconstruct EEAs with respect to heterogenous sets of behavior. The ethically relevant medical question is about who decides on what abilities the individual should be constituted of. What I am proposing is to use a kind of ability as the second order ability to determine the other kind of abilities. This ability in this regard is the ability to determine your own environment. This comes in two distinct elements, one is epistemic determination, and the other is physical determination. The distinction between the environment and the organism, although not unique to niche construction or evolutionary medicine, enables the patients to determine what is relevant within their own environments.

## Data Availability

Not applicable.

## References

[CR1] Altinok, O. 2022. Darwinize it two times: On the possibilities of extending evolutionary medicine through new developments in evolutionary theory. *Azimuth* 19 (1): 197–210.

[CR2] Altinok, O. 2023. Evolution and evolutionary medicine in disease. In *Conceptual and ethical challenges of evolutionary medicine ethics of science and technology assessment*, vol. 53. Cham: Springer.

[CR3] Amundson, R. 1992. Disability, handicap, and the environment. *Journal of Social Philosophy* 23 (1): 105–19. 10.1111/j.1467-9833.1992.tb00489.x. 11659501 10.1111/j.1467-9833.1992.tb00489.x

[CR4] Anderson, W. 2006. *Colonial pathologies*. Durham: Duke University Press.

[CR94] Badyaev, A.V., and T. Uller. 2009. Parental effects in ecology and evolution: Mechanisms, processes and implications. *Philosophical Transactions of the Royal Society B* 364 (1520): 1169–1177. 10.1098/rstb.2008.0302 PMC2666689 19324619

[CR5] Barnes, C., G. Mercer, and T. Shakespeare. 1999. *Exploring Disability: A sociological introduction*. Cambridge: Polity Press.

[CR6] Barnes, C. 2003. The social model of disability: A sociological phenomenon ignored by sociologists. In *The disability reader (2nd edition)*, 266–273. London: Routledge.

[CR7] Baedke, J. 2017. Expanding views of evolution and causality. *Journal for General Philosophy of Science* 48 (4): 591–594. 10.1007/s10838-017-9371-2. 10.1007/s10838-017-9371-2

[CR8] Baedke, J. 2018. *Above the gene, beyond biology: Toward a philosophy of epigenetics*. Pittsburgh: University of Pittsburgh Press.

[CR9] Barton, L. 1996. *Disability and society: Emerging issues and insights*. New York: Longman.

[CR93] Bonduriansky, R. 2012. Rethinking heredity, again. *Trends in Ecology & Evolution* 27 (6): 330–336. 22445060 10.1016/j.tree.2012.02.003

[CR10] Boorse, C. 1975. On the distinction between disease and illness. *Philosophy & Public Affairs* 5: 49–68.

[CR11] Boorse, C. 1976. Wright on functions. *The Philosophical Review* 85 (1): 70–86. 10.2307/2184255

[CR12] Boorse, C. 1977. Health as a Theoretical Concept. *Philosophy of Science* 44 (4): 542–573. 10.1086/288768. 10.1086/288768

[CR13] Boorse, C. 2014. A second rebuttal on health. *Journal of Medicine and Philosophy* 39 (6): 683–724. 25398760 10.1093/jmp/jhu035

[CR14] Bowlby, J. 1969. *Attachment and loss*. New York: Basic Books.

[CR15] Brigandt, I. 2010. The epistemic goal of a concept: Accounting for the rationality of semantic change and variation. *Synthese* 177 (1): 19–40. 10.1007/s11229-009-9623-8. 10.1007/s11229-009-9623-8

[CR84] Brigandt, I. 2014. From developmental constraint to evolvability: How concepts figure in explanation and disciplinary identity. In *Conceptual change in biology: Scientific and philosophical perspectives on evolution and development*, 305–325. Dordrecht: Springer.

[CR16] Broadbent, A. 2019. Health as a secondary property. *The British Journal for the Philosophy of Science* 70 (2): 609–627. 10.1093/bjps/axx014. 31086424 10.1093/bjps/axx014 PMC6505580

[CR100] Broom, D.M. 1993. A usable definition of animal welfare. *Journal of Agricultural and Environmental Ethics* 6 (Suppl 2): 15–25.

[CR17] Canguilhem, G. 1991. *The normal and the pathological*. Trans. C.R. Fawcett. New York: Zone Books.

[CR18] Cooper, I.R. 2020. The concept of disorder revisited: Robustly value-laden despite change. *Aristotelian Society Supplementary* 94 (1): 141–161. 10.1093/arisup/akaa010. 10.1093/arisup/akaa010

[CR19] Cournoyea, M. 2018. *Medical explanations in evolutionary medicine, network medicine, and medically unexplained physical symptoms* (Doctoral Dissertation).

[CR20] Cournoyea, M., and A.G. Kennedy. 2014. Causal explanatory pluralism and medically unexplained physical symptoms. *Journal of Evaluation in Clinical Practice* 20 (6): 928–933. 10.1111/jep.12238. 25346453 10.1111/jep.12238

[CR92] Danchin, É., A. Charmantier, F.A. Champagne, A. Mesoudi, B. Pujol, and S. Blanchet. 2011. Beyond DNA: Integrating inclusive inheritance into an extended theory of evolution. *Nature Reviews Genetics* 12 (7): 475–486. 10.1038/nrg3028 21681209

[CR21] Engel, G.L. 1977. The need for a new medical model: A challenge for biomedicine. *Science*. 10.1126/science.847460. 10.1126/science.847460 847460

[CR24] Feinstein, A.R. 1976. *Clinical judgment*. Philadelphia: Williams & Wilkins.

[CR25] Foucault, M. 1975. Discipline and punish. A. Sheridan, Tr., Paris, FR, Gallimard.

[CR26] Firth, S. 2023. The picture theory of disability. *Cambridge Quarterly of Healthcare Ethics*. 10.1017/S0963180123000154. 10.1017/S0963180123000154 37017202

[CR27] Griffiths, P.E., and J. Matthewson. 2018. Evolution, dysfunction, and disease: A reappraisal. *The British Journal for the Philosophy of Science*. 10.1093/bjps/axw021. 10.1093/bjps/axw021

[CR28] Grunspan, D.Z., R.M. Nesse, M.E. Barnes, and S.E. Brownell. 2018. Core principles of evolutionary medicine: A Delphi study. *Evolution, Medicine, and Public Health* 2018 (1): 13–23. 10.1093/emph/eox025. 29493660 10.1093/emph/eox025 PMC5822696

[CR29] Gustavasson, A. 2004. The role of disability research—Springboard or straight-jacket? *Scandinavian Journal of Disability Research* 6 (1): 55–70. 10.1080/15017410409512639. 10.1080/15017410409512639

[CR86] Hofmann, B. 2001. Complexity of the concept of disease as shown through rival theoretical frameworks. *Theoretical Medicine and Bioethics* 22: 211–236. 10.1023/A:1011416302494. 11499496 10.1023/A:1011416302494

[CR30] Hofmann, B. 2002. On the triad disease, illness and sickness. *Journal of Medicine and Philosophy* 27 (6): 651–673. 10.1076/jmep.27.6.651.13793. 12607162 10.1076/jmep.27.6.651.13793

[CR31] Jablonka, E., and M.J. Lamb. 2005. *Evolution in four dimensions: Genetic, epigenetic, behavioral, and symbolic variation in the history of life*. Cambridge: MIT Press.

[CR32] Jablonka, E., and G. Raz. 2009. Transgenerational epigenetic inheritance: Prevalence, mechanisms, and implications for the study of heredity and evolution. *The Quarterly Review of Biology*. 10.1086/598822. 10.1086/598822 19606595

[CR33] Kaiser, M.I., and R. Trappes. 2021. Broadening the problem agenda of biological individuality: Individual differences, uniqueness and temporality. *Biology and Philosophy* 36: 15. 10.1007/s10539-021-09791-5. 10.1007/s10539-021-09791-5

[CR34] Kass, L. 1975. The end of medicine and the pursuit of health. *The Public Interest* 40: 11–42. 11662217

[CR97] Kearney, M. 2006. Habitat, environment and niche: What are we modelling? *Oikos* 115 (1): 186–191. 10.1111/j.2006.0030-1299.14908.x

[CR35] Kingma, E. 2014. Naturalism about health and disease: Adding nuance for progress. *Journal of Medicine and Philosophy* 39 (6): 590–608. 10.1093/jmp/jhu037. 25376497 10.1093/jmp/jhu037

[CR36] Kingma, E. 2007. What is it to be healthy? *Analysis* 67 (2): 128–133. 18270546 10.1093/analys/67.2.128 PMC2239248

[CR91] Kranke, N. 2022. Two kinds of historical explanation in evolutionary biology. *Biology & Philosophy* 37 (3): 17. 10.1007/s10539-022-09848-z

[CR37] Krohs, U. 2022. Darwin’s empirical claim and the janiform character of fitness proxies. *Biology & Philosophy* 37 (2): 15. 10.1007/s10539-022-09847-0

[CR38] Laland, K., B. Matthews, and M.W. Feldman. 2016. An introduction to niche construction theory. *Evolutionary Ecology* 30: 191–202. 27429507 10.1007/s10682-016-9821-z PMC4922671

[CR39] Laland, K.N., K. Sterelny, J. Odling-Smee, W. Hoppitt, and T. Uller. 2011. Cause and effect in biology revisited: Is Mayr’s proximate-ultimate dichotomy still useful? *Science (New York, NY)* 334 (6062): 1512–1516. 10.1126/science.1210879. 10.1126/science.1210879 22174243

[CR40] Laland, K.N., T. Uller, M.W. Feldman, K. Sterelny, G.B. Müller, A. Moczek, E. Jablonka, and J. Odling-Smee. 2015. The extended evolutionary synthesis: Its structure, assumptions and predictions. *Proceedings Biological Sciences* 282 (1813): 20151019. 10.1098/rspb.2015.1019. 26246559 10.1098/rspb.2015.1019 PMC4632619

[CR41] Laland, K., T. Uller, M. Feldman, K. Sterelny, G.B. Müller, A. Moczek, E. Jablonka, J. Odling-Smee, G.A. Wray, H.E. Hoekstra, D.J. Futuyma, R.E. Lenski, T.F.C. Mackay, D. Schluter, and J.E. Strassmann. 2014. Does evolutionary theory need a rethink? *Nature* 514 (7521): 161–164. 10.1038/514161a. 25297418 10.1038/514161a

[CR42] Lemoine, M. 2013. Defining disease beyond conceptual analysis: An analysis of conceptual analysis in philosophy of medicine. *Theoretical Medicine and Bioethics* 34 (4): 309–325. 10.1007/s11017-013-9261-5. 23868106 10.1007/s11017-013-9261-5

[CR46] Margolis, J. 1976. The concept of disease. *Journal of Medicine and Philosophy* 1 (3): 238–255. 10.1093/jmp/1.3.238. 10.1093/jmp/1.3.238

[CR45] Matthews, B., L. De Meester, C.G. Jones, et al. 2014. Under niche construction: An operational bridge between ecology, evolution and ecosystem science. *Ecological Monographs* 84 (2): 245–263. 10.1890/13-0953.1

[CR44] Mendelson, G. 2003. Homosexuality and psychiatric nosology. *Australian and New Zealand Journal of Psychiatry* 37 (6): 678–683. 10.1111/j.1440-1614.2003.01273.x. 14636381 10.1111/j.1440-1614.2003.01273.x

[CR88] Millikan, R. 1984. *Language, thought and other biological categories*. Cambridge: MIT Press.

[CR47] Millikan, R. 1989. In defense of proper functions. *Philosophy of Science* 56 (2): 288–302. 10.1086/289488

[CR85] Morar, N., and J.A. Skorburg. 2018. Bioethics and the hypothesis of extended health. *Kennedy Institute of Ethics Journal* 28 (3): 341–376. 30369509 10.1353/ken.2018.0020

[CR49] Müller, G.B., and M. Pigliucci. 2010. Extended synthesis: Theory expansion or alternative? *Biological Theory* 5 (3): 275–276. 10.1162/BIOT_a_00050

[CR87] Neander, K. 1991. The teleological notion of ‘function.’ *Australasian Journal of Philosophy* 69 (4): 454–468. 10.1080/00048409112344881

[CR50] Nesse, R.M. 2001a. On the difficulty of defining disease: A Darwinian perspective. *Medicine, Health Care and Philosophy* 4 (1): 37–46. 11315418 10.1023/A:1009938513897

[CR51] Nesse, R.M. 2001b. The smoke detector principle. *Annals of the New York Academy of Sciences* 935 (1): 75–85. 10.1111/j.1749-6632.2001.tb03472.x. 11411177 10.1111/j.1749-6632.2001.tb03472.x

[CR52] Nesse, R.M. 2007a. Evolution is the scientific foundation for diagnosis: Psychiatry should use it. *World Psychiatry* 6 (3): 160–161. 18188435 PMC2174585

[CR53] Nesse, R.M. 2007. The importance of evolution in medicine. In *Evolutionary medicine and health: New perspectives*, ed. W.R. Trevathan, E.O. Smith, and J. McKenna, 416–433. Oxford: Oxford University Press.

[CR54] Nesse, R.M. 2011. Why has natural selection left us so vulnerable to anxiety and mood disorders? *Canadian Journal of Psychiatry* 56 (12): 705–706. 10.1177/070674371105601201. 22152638 10.1177/070674371105601201

[CR90] Nesse, R.M. 2019. *Good reasons for bad feelings: Insights from the frontier of evolutionary psychiatry*. New York: Penguin.

[CR56] Nordby, H. 2019. Who are the rightful owners of the concepts disease, illness and sickness? A pluralistic analysis of basic health concepts. *Open Journal of Philosophy* 09 (04): 470–492. 10.4236/ojpp.2019.94029. 10.4236/ojpp.2019.94029

[CR57] Nordenfelt, L. 1986. Health and disease: Two philosophical perspectives. *Journal of Epidemiology and Community Health* 40 (4): 281–284. 3655618 10.1136/jech.40.4.281 PMC1052546

[CR58] Nordenfelt, L. 1993. On the notions of disability and handicap. *International Journal of Social Welfare* 2 (1): 17–24.

[CR99] Nordenfelt, L. 2017. On concepts of positive health. *Handbook of the Philosophy of Medicine*, 29–43. ISO 690.

[CR59] Odling-Smee, F.J. 1988. Niche constructing phenotypes. In *The role of behavior in evolution*, ed. J. Plotkin. Cambridge: MIT Press.

[CR60] Odling-Smee, F.J., K.N. Laland, and M.W. Feldman. 2003. Niche construction: The neglected process in evolution. In *Monographs in population biology*, vol. 37. Princeton: Princeton University Press.

[CR61] Oliver, M. 1990. *The politics of disablement*. London: Macmillan.

[CR98] Pocheville, A. 2015. The ecological niche: History and recent controversies. *Handbook of evolutionary thinking in the sciences*, 547–586.

[CR83] Putnam, H. 1975. *Mind, language and reality: Philosophical papers*, vol. 2. Cambridge: Cambridge University Press.

[CR62] Sarto-Jackson, I. 2018. Time for a change: Topical amendments to the medical model of disease. *Biological Theory* 13 (1): 29–38. 10.1007/s13752-017-0289-z

[CR96] Schoener, T.W. 1989. Food webs from the small to the large. *Ecology* 70 (6): 1559–1589. 10.2307/1938088

[CR63] Shakespeare, T. 2006. The social model of disability. *The Disability Studies Reader* 2: 197–204.

[CR64] Shakespeare, T. 2013. *Disability rights and wrongs revisited*. London: Routledge.

[CR65] Stearns, S.C. 2012. Evolutionary medicine: Its scope, interest and potential. *Proceedings Biological Sciences* 279 (1746): 4305–4321. 10.1098/rspb.2012.1326. 22933370 10.1098/rspb.2012.1326 PMC3479795

[CR66] Stempsey, W.E. 2000. *Disease and diagnosis: Value-dependent realism*. Dordrecht: Springer.

[CR69] Trappes, R. 2021a. Personality, persistence, and the perplexing uniqueness of biological individuals.

[CR70] Trappes, R. 2021b. Defining the niche for niche construction: Evolutionary and ecological niches. *Biology and Philosophy* 36: 31. 10.1007/s10539-021-09805-2. 10.1007/s10539-021-09805-2

[CR71] Trappes, R., B. Nematipour, and U. Krohs. 2022. Introduction to niches and mechanisms in ecology and evolution. *Biology and Philosophy* 37: 55. 10.1007/s10539-022-09890-x. 10.1007/s10539-022-09890-x

[CR72] Tresker, S. 2020. Theoretical and clinical disease and the biostatistical theory. *Studies in History and Philosophy of Science Part C: Studies in History and Philosophy of Biological and Biomedical Sciences* 82: 101249. 10.1016/j.shpsc.2019.101249 32008896

[CR73] Trevathan, W. 2018. Evolutionary medicine. In *The international encyclopedia of biological anthropology*, ed. Wenda Trevathan, 1–4. Hoboken: Wiley.

[CR74] Trevathan, W.R., E.O. Smith, and J.J. McKenna. 2008. *Evolutionary medicine and health: New perspectives*. Oxford: Oxford University Press.

[CR75] Uller, T., N. Feiner, R. Radersma, I.S.C. Jackson, and A. Rago. 2020. Developmental plasticity and evolutionary explanations. *Evolution & Development* 22 (1–2): 47–55. 10.1111/ede.12314. 31535438 10.1111/ede.12314

[CR76] Wakefield, J.C. 1992. Disorder as harmful dysfunction: A conceptual critique of DSM-III-R’s definition of mental disorder. *Psychological Review* 99 (2): 232. 1594724 10.1037/0033-295X.99.2.232

[CR77] Wakefield, J.C. 2011. Darwin, functional explanation, and the philosophy of psychiatry. In *Maladapting minds: Philosophy, psychiatry, and evolutionary theory*, ed. P.R. Adriaens and A. De Block. New York: Oxford University Press.

[CR79] White, K. 2008. *An introduction to the sociology of health and illness*. New York: SAGE.

[CR80] Williams, G.C., and R.M. Nesse. 1991. The dawn of Darwinian medicine. *The Quarterly Review of Biology* 66 (1): 1–22. 10.1086/417048. 2052670 10.1086/417048

[CR95] Wright, L. 1973. Functions. *Philosophical Review* 82: 139–168.

[CR81] Zampieri, F. 2009. Medicine, evolution, and natural selection: An historical overview. *The Quarterly Review of Biology* 84 (4): 333–355. 10.1086/648122. 20039527 10.1086/648122

[CR82] Zinsstag, J., E. Schelling, L. Crump, M. Whittaker, and C. Stephen, eds. 2020. *One health: The theory and practice of integrated health approaches*. Egham: CABI.

